# Hemosuccus Pancreaticus: A Mysterious Cause of Gastrointestinal Bleeding

**DOI:** 10.14740/gr596w

**Published:** 2014-03-14

**Authors:** Rohan Mandaliya, Benjamin Krevsky, Abhinav Sankineni, Kiley Walp, Oliver Chen

**Affiliations:** a Department of Internal Medicine, Abington Memorial Hospital, Abington, PA, USA; b Gastroenterology Section, Department of Medicine, Temple University School of Medicine, Philadelphia, PA, USA; c Department of Radiology, Temple University School of Medicine, Philadelphia, PA, USA

**Keywords:** Hemosuccus pancreaticus, Upper gastrointestinal bleeding, Pancreatitis, Pseudoaneurysm, Angiography, Endoscopy

## Abstract

Hemosuccus pancreaticus (bleeding from the pancreatic duct into the gastrointestinal tract via the ampulla of Vater) is a rare, potentially life-threatening and obscure cause of upper gastrointestinal bleeding. It is caused by rupture of the psuedoaneurysm of a peripancreatic vessel into pancreatic duct or pancreatic psuedocyst in the context of pancreatitis or pancreatic tumors. It can pose a significant diagnostic and therapeutic dilemma due to its anatomical location and that bleeding into the duodenum is intermittent and cannot be easily diagnosed by endoscopy. A 61-year-old female with HIV and alcoholism presented with 3 weeks of intermittent abdominal pain and melena. Examination revealed hypotension with pallor and mild epigastric tenderness. She was found to have severe anemia and a high serum lipase. It was decided to perform a contrast-enhanced computed tomography (CT) scan that demonstrated a hemorrhagic pancreatic pseudocyst with possible active bleeding into the cyst. An emergent angiogram showed a large pseudoaneurysm of the pancreaticoduodenal artery that was successfully embolized. Subsequent endoscopy showed blood near ampulla of Vater confirming the diagnosis of hemosuccus pancreaticus. Thus the bleeding pseudocyst was communicating with pancreatic duct. The patient had no further episodes of gastrointestinal bleeding. Hemosuccus pancreaticus should be considered in patients with intermittent crescendo-decrescendo abdominal pain, gastrointestinal bleeding and a high serum lipase. Contrast-enhanced CT scan can be an excellent initial diagnostic modality and can lead to prompt angiography for embolization of the bleeding pseudoaneurysm and can eliminate the need for surgery.

## Introduction

Hemosuccus pancreaticus is described as bleeding from the ampulla of Vater via the pancreatic duct [1, 2]. It is one of the least frequent causes of upper gastrointestinal bleeding and is most often caused by chronic pancreatitis, pancreatic pseudocysts or pancreatic tumors. The term “hemosuccus pancreaticus” was first coined by Sandblom in 1970 [2]. In the medical literature, hemosuccus pancreaticus is mostly limited to limited case reports. The purpose of our study is: 1) to review a single case demonstrating the clinical entity, “hemosuccus pancreaticus”; 2) to highlight the challenges in the diagnosis and management of this potentially life-threatening condition and propose an initial screening diagnostic modality based on specific clinical presentation.

## Case Report

A 61-year-old female with a history of HIV (on efavirenz, emtricitabine and tenofovir) and chronic alcoholism, presented to the hospital with epigastric pain and melena. The patient was having intermittent epigastric abdominal pain for about 3 weeks which waxed and waned without intervention. The patient was noticed dark tarry stools. There was no prior history of liver disease or gastrointestinal bleeding. On examination, the patient was hypotensive with a blood pressure of 90/60 mmHg with a heart rate of 100 beats per minute. The patient was pale with mild epigastric tenderness present on palpation without any distension or organomegaly. Rectal examination revealed black stools. Initial laboratory studies revealed hemoglobin of 4.8 g/dL. Serum lipase was elevated to 870 U/L (normal range 10 - 140 U/L) as was serum amylase 1,000 U/L (normal range 40 - 140 U/L), with normal liver function tests and electrolytes. There was no coagulopathy. The CD4 count was 400 cells/mm^3^.

**Figure 1:**
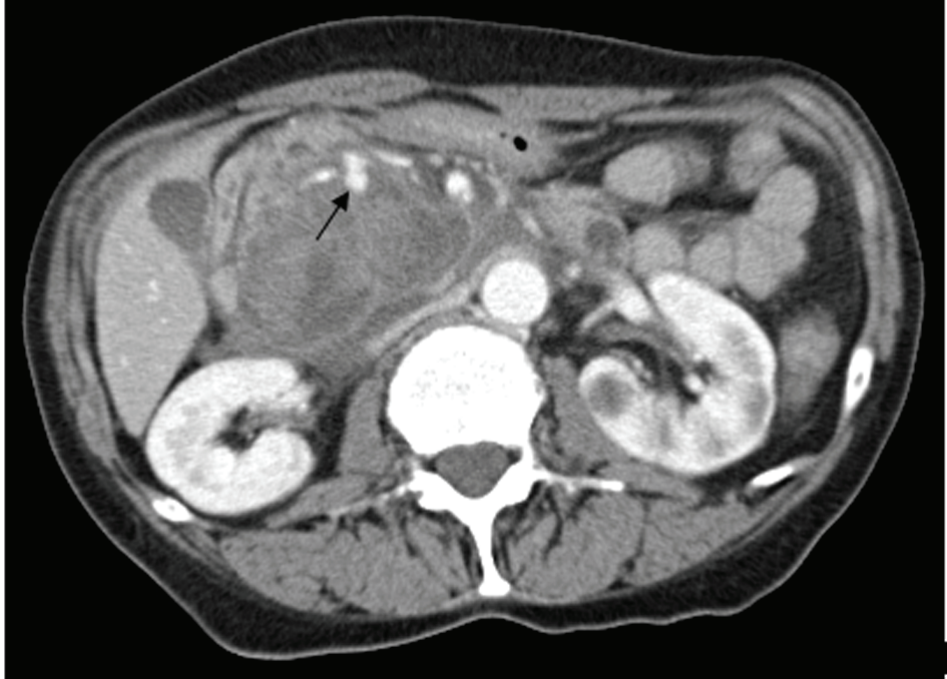
Contrast-enhanced CT scan of the abdomen reveals a 5 × 6 × 7 cm complex cystic mass in the region of uncinate process of pancreatic head with an enhancing capsule and a small hyperdensity consistent with pseudoaneurysm of a peripancreatic vessel with active bleeding into the pancreatic pseudocyst.

**Figure 2:**
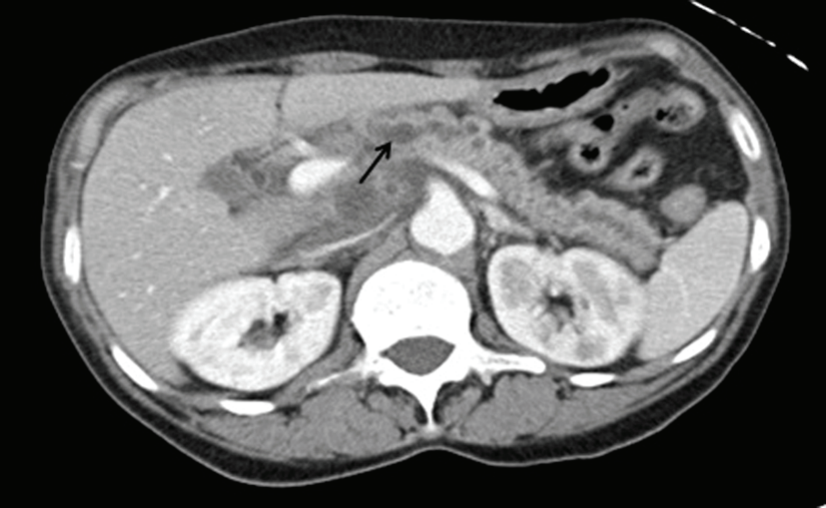
Contrast-enhanced CT scan of the abdomen reveals dilated pancreatic duct possibly filled with blood.

**Figure 3:**
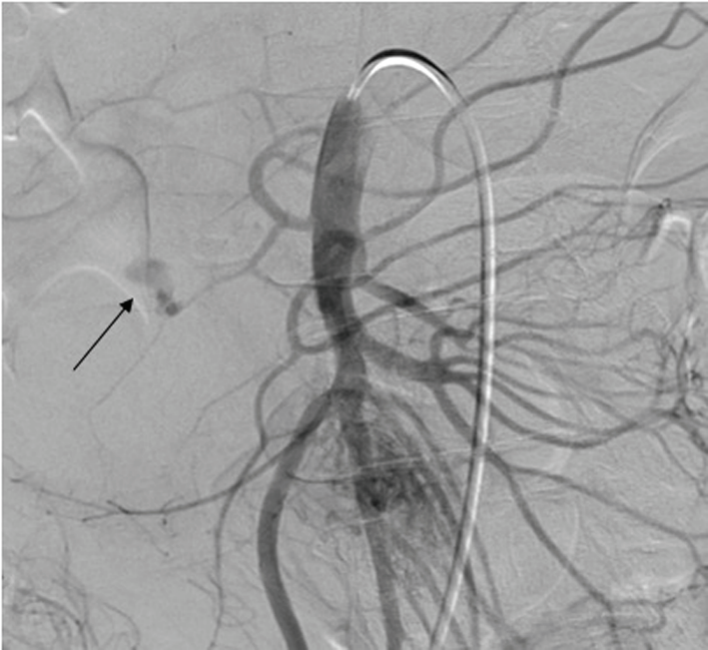
Abdominal angiography demonstrates an actively bleeding large pseudoaneurysm in the peripancreatic vessel arcade likely in the branch of pancreaticoduodenal artery.

**Figure 4:**
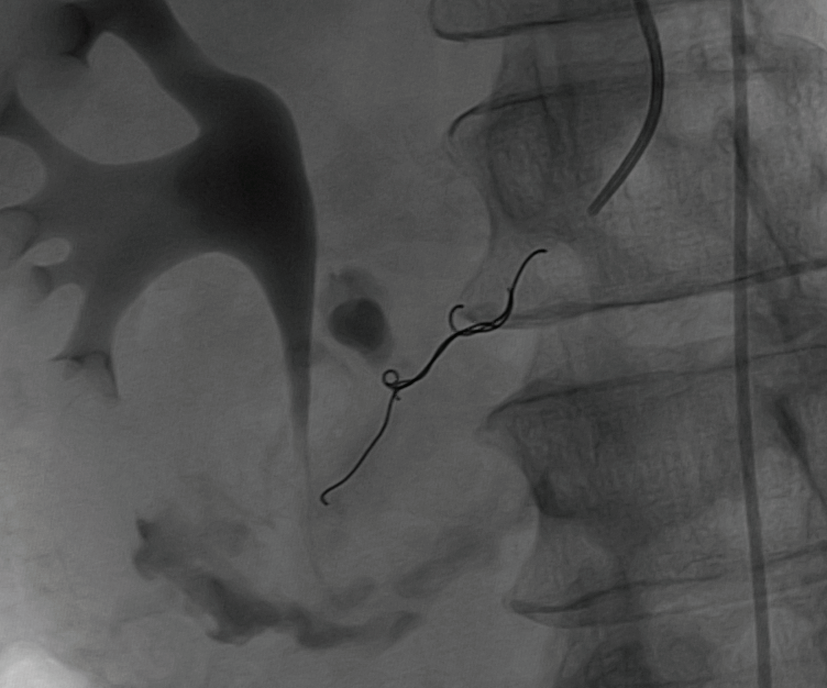
Post embolization angiogram shows embolization of the pancreaticoduodenal artery with resolution of contrast opacification of the bleeding pseudoaneurysm.

After initial resuscitation, it was decided to perform an emergent contrast-enhanced computed tomography (CT) of the abdomen in lieu of a diagnostic endoscopy because of the high serum lipase. It revealed a 5 × 6 × 7 cm complex cystic mass in the region of uncinate process of pancreatic head with an enhancing capsule and a small hyperdensity consistent with pseudoaneurysm of a peripancreatic vessel with active bleeding into the pancreatic pseudocyst (Fig. 1). It also demonstrated a dilated pancreatic duct (Fig. 2). An angiography was performed that demonstrated a large pseudoaneurysm in the peripancreatic vessel arcade likely in the branch of pancreaticoduodenal artery (Fig. 3). Subsequently a microcoil and glue embolization of the pancreaticoduodenal artery was performed, with resolution of contrast opacification of the pseudoaneurysm (Fig. 4).

**Figure 5:**
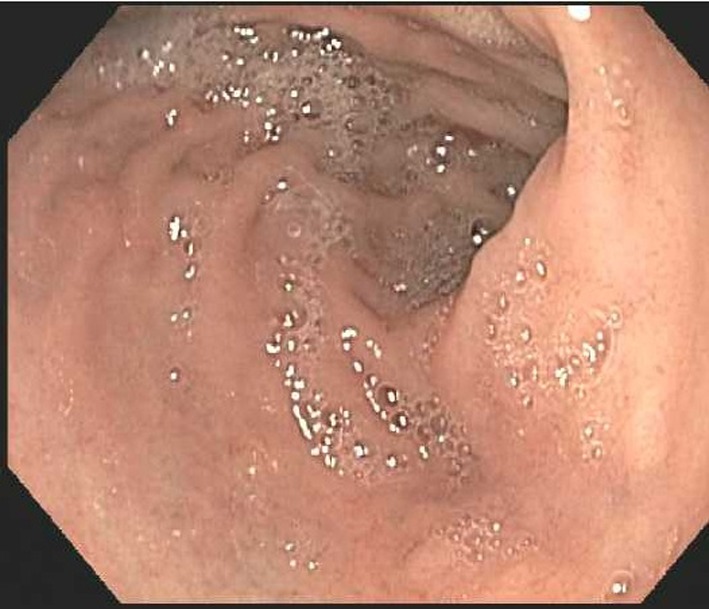
Esophagogastroduodenoscopy shows with no bleeding source in the stomach.

**Figure 6:**
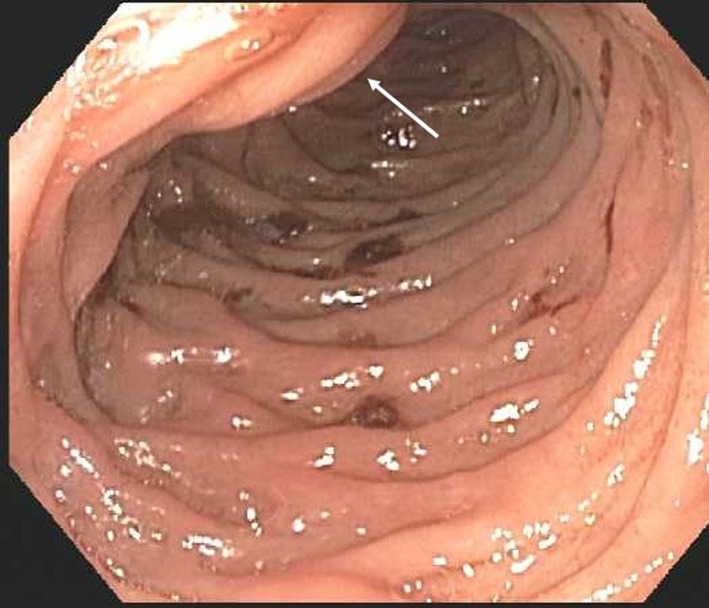
Esophagogastroduodenoscopy shows old blood in the duodenum near the ampulla of Vater, which is likely the source of bleeding into gastrointestinal tract. Arrow pointing towards the ampulla of Vater.

An esophagogastroduodenoscopy (EGD) was then performed to further evaluate the cause of melena. There was no blood in the stomach (Fig. 5). It demonstrated some old blood in duodenum near the ampulla of Vater (Fig. 6). No active source of bleeding was identified. It appeared that the blood was intermittently passing out of the ampulla of Vater due to the communication between hemorrhagic pseudocyst and pancreatic duct. This condition is called hemosusccus pancreaticus.

The patient did not have further episodes of bleeding or abdominal pain. The patient was discharged with follow-up imaging a few weeks later.

## Discussion

Hemosuccus pancreaticus is a rare and potentially life-threatening obscure cause of upper gastrointestinal bleeding. It is described as bleeding from the ampulla of Vater via the pancreatic duct. It is one of least frequent cause of upper gastrointestinal bleeding and is most often caused by chronic pancreatitis, pancreatic tumors and sometimes pancreatic pseudocysts [1-4]. The condition is often difficult to diagnose at an early stage because of its rarity, anatomical location and the fact that the bleeding is often intermittent and cannot be easily diagnosed by EGD [1-5].

Hemosuccus pancreaticus has been estimated to occur in about one in 1,500 cases of gastrointestinal bleeding [6]. Causes include pancreatitis, vascular malformation [7], pancreatic tumors (pancreatic carcinoma, serous cystic neoplasm and neuroendocrine tumors) [8-10], pancreas divisum with chronic pancreatitis [11] and iatrogenic (two cases reported after endoscopic ultrasound (EUS)-fine needle aspiration) [12, 13].

It is most frequently caused by the rupture of a pseudoaneurysm of the peripancreatic arteries associated with acute and chronic pancreatitis [14]. The arteries involved in the gastrointestinal hemorrhage in the order of frequency include: splenic, gastroduodenal, pancreaticoduodenal, gastric and hepatic arteries [15, 16]. The pseudoaneurysm can rupture into the gastrointestinal tract, peritoneal cavity, pancreatic parenchyma or pancreatic pseudocyst [17, 18]. In our patient the pseudoaneurysm ruptured into pancreatic pseudocyst which communicated with the pancreatic duct.

Patients with hemosuccus pancreaticus usually present with abdominal pain, gastrointestinal hemorrhage and high amylase levels [19]. Abdominal pain and bleeding are usually intermittent. The abdominal pain is thought to be caused by increased intraductal pressure due to distension of main duct with blood [6]. Caly et al emphasized the fact that the characteristic crescendo-decrescendo nature of pain is found in approximately half of the patients, as a result of sequential distension and decompression of the pancreatic duct by blood and clot [20]. Gastrointestinal bleeding, which can be melena, hemetemesis or rarely hematochezia, usually follows in a day or two, relieving the pain. Also, the intermittent nature of bleeding is caused by the formation and dissolution of a clot in the main pancreatic duct or pseudocyst [5]. An elevated serum amylase is usually seen due to increased intraductal/intracystic pressure [19].

The diagnosis of the condition can be extremely difficult due to its rarity, anatomical location and intermittent symptoms. Abdominal pain, gastrointestinal hemorrhage and hyperamylasemia should raise a high level of suspicion of hemosuccus pancreaticus as a possibility in the setting of history of chronic pancreatitis, pancreatic tumors and pancreatic vascular diseases. Episodes of recurrent epigastric abdominal pain radiating to the back and resolving spontaneously are usually followed by melena.

It is important to know the other two rare causes of extra luminal source of gastrointestinal bleeding into the duodenum which can be missed easily and can be life-threatening. These include hemobilia and primary aortoenteric fistula. These are the rare causes of obscure gastrointestinal bleeding and it is important to differentiate one condition from the other. Table 1 compares the clinical characteristics and management of these three different conditions.

**Table 1:** Comparison of the Three Rare Causes of Upper Gastrointestinal Bleeding

	Hemosuccus pancreaticus	Hemobilia	Primary aortoenteric fistula
Definition	Bleeding from the pancreatic duct into duodenum via the ampulla of Vater.	Bleeding from the biliary tract into duodenum via ampulla of Vater.	Bleeding from the aorta into the duodenum (most common) via fistula between aorta and duodenum.
Source	Pancreas, pancreatic pseudocyst.	Intrahepatic, extrahepatic like gall bladder or bile duct.	Aorta.
Bleeding vessel	Peripancreatic vessels like splenic, gastroduodenal, pancreaticoduodenal, splenic and sometimes hepatic artery.	Hepatic artery, branch of right or left hepatic artery.	Aorta.
Site of bleeding into the gastrointestinal tract	Second part of duodenum (ampulla of Vater).	Second part of duodenum (ampulla of Vater).	Third part of duodenum (most common).
Classic triad	Abdominal pain, gastrointestinal hemorrhage and hyperamylasemia.	Abdominal pain, gastrointestinal hemorrhage and jaundice.	Abdominal pain, gastrointestinal hemorrhage and pulsatile abdominal mass.
Characteristic picture	Crescendo-decrescendo abdominal pain followed by hemorrhage with a repeat cycle of pain followed by hemorrhage.	Abdominal pain and hemorrhage usually with a recent history of instrumentation.	“Herald” hemorrhage followed hours, days, or weeks later by catastrophic hemorrhage.
Causes	Chronic pancreatitis, pancreatic pseudocyst, pancreatic tumors, iatrogenic like EUS/ERCP, vascular malformations, and so on.	Iatrogenic (liver biopsy, percutaneous transhepatic cholangiography, instrumentation, and so on), trauma, hepatobiliary malignancy, inflammation (cholangitis, vasculitis, gallstone disease), parasitic infection, vascular malformation, and so on.	Aortic aneurysm (majority atherosclerotic, followed by mycotic aneurysms), septic aortitis, radiation, carcinoma, ulcers, and so on.
Diagnostic test	Contrast CT scan very helpful, EGD (diagnostic in 30%), angiography.	Contrast CT scan very helpful, EGD (diagnostic in 12%), ERCP in some cases, technitium red cell scan in some cases, angiography.	Contrast CT scan the most diagnostic. EGD in initial phase when bleeding is herald. Angiography usually not performed as most patients are critically ill when considered for angiography.
Treatment	Transcatheter arterial embolization of the pancreatic vessel, Surgery is TAE fails; includes ligation of bleeding vessel, excision of aneurysm, central/distal pancreatectomy.	Transcatheter arterial embolization of hepatic artery (first approach). Surgery if TAE fails; includes ligation of bleeding vessel, excision of aneurysm. Further options depend on site of bleeding; partial hepatectomy, cholecystectomy.	Emergent laparotomy. Debridement of diseased aorta and repair with prosthetic graft along with primary repair of the gastrointestinal tract.

The various diagnostic modalities that can aid in the diagnosis of hemosuccus pancreaticus include contrast-enhanced CT, esophagogastroduodenoscopy, selective angiography and sometimes EUS and endoscopic retrograde cholangio pancreatography (ERCP). Hemorrhage from the ampulla is not commonly identified at endoscopy due to intermittent nature of bleeding and the suboptimal view of the ampulla with a forward viewing gastroscope. Endoscopy can detect active bleeding via the papilla in only 30% of the patients [8]. In rare instances, endoscopy demonstrates bleeding from the ampulla [21, 22]. Thus, endoscopy is usually not sufficient to make an initial precise diagnosis and a negative endoscopy does not exclude the possibility of hemosusccus pancreaticus. However endoscopy is essential in ruling out other causes of upper gastrointestinal bleeding such as peptic ulcer, erosive gastritis, varices, and so on.

Contrast-enhanced CT scan of the abdomen is an excellent modality for demonstrating the pancreatic pathology and can also demonstrate features of chronic pancreatitis, psuedocysts and pseudoaneurysms. It may show simultaneous opacification of an aneurysmal artery and pseudocyst or penetration of contrast within a pseudocyst after the arterial phase. When identified, these findings should lead to an urgent angiography for definitive diagnosis as well as therapeutic intervention as in the current case. Thus CT scan remains an important diagnostic modality which may help for early therapeutic intervention. Finally, angiography remains the gold standard for diagnosis and therapy. It identifies the causative artery and helps to delineate arterial anatomy for therapeutic intervention [4, 5, 15]. The sensitivity of angiography is usually greater than 90% [23]. In a recent retrospective study at a tertiary care hospital in patients with hemosuccus pancreaticus, ultrasound yielded a positive result in 38% of the patients followed by endoscopy (51%), contrast-enhanced CT (90%) and angiography (89%) [4].

The treatment of hemosuccus pancreaticus should eradicate the source of bleeding completely. There are two potential approaches: interventional radiological procedures and surgery. If the source of hemorrhage is found by angiography then interventional radiographic procedures are the first choice for initial management with immediate good results in 79-100% of the cases and an overall success rate of 67% [23, 24]. The techniques for intervention include embolization via prosthetic material, balloon tamponade and stent placement. Coil embolization is the most frequently described technique. It stimulates thrombus formation in the pseudoaneurysm. However it also occludes the artery and ischemia can develop in the tissue supplied by the artery if the collateral supply is poor. Balloon tamponade and stent placement can be used as a bridge to elective surgery. Benz et al described for the first time successful implantation of an uncoated metal Palmaz stent across the aneurysmal segment of the splenic artery [25]. The recurrence rate of bleeding after angiographic embolization is around 30% [23].

Surgical treatment is indicated when there is uncontrolled bleeding, persistent shock, failure of embolization, rebleeding after embolization, or when initial angiography shows no abnormal findings. The various surgical procedures include distal pancreatectomy and splenectomy, central pancreatectomy, intracystic ligation of the blood vessel, aneurysm ligation and bypass graft [4, 23]. During the surgical procedure, it is sometimes difficult to confirm the source of bleeding and to determine the resecting line of the pancreas. Intraoperative ultrasonography and intraoperative pancreatoscopy can be performed to determine the origin of bleeding and thus determine the extent of pancreatic resection [26]. Most surgical procedures have shown success rates of 70-85%, at the same time operative mortality rates of 10-50% have been reported in the literature [21, 23]. The rate of rebleeding after surgery is around 0-5% [23].

Will et al reported for the first time a novel technique of EUS guided angiotherapy in treating hemosuccus pancreaticus. It can be an important diagnostic and therapeutic tool in selected candidates who do not have an angiographic evidence of bleeding, in whom contrast cannot be administered, or who are poor candidates for surgery [27]. However the safety and efficacy of such innovative techniques should be confirmed.

### Conclusions

Hemosuccus pancreaticus is a life-threatening condition and should be considered in patients with abdominal pain, gastrointestinal hemorrhage and high serum lipase. Although the early diagnosis is challenging, use of appropriate diagnostic studies plays a significant role in reducing mortality. Contrast-enhanced CT scan of the abdomen can be an excellent initial diagnostic test with a high sensitivity, can locate the source of bleeding and lead to prompt angiographic embolization of the bleeding pseudoaneurysm. EUS can be an innovative approach for the diagnosis and treatment of patients in whom contrast cannot be administered; however, its safety and efficacy need to be confirmed by future studies.
